# QF2011: a protocol to study the effects of the Queensland flood on pregnant women, their pregnancies, and their children's early development

**DOI:** 10.1186/s12884-015-0539-7

**Published:** 2015-05-06

**Authors:** Suzanne King, Sue Kildea, Marie-Paule Austin, Alain Brunet, Vanessa E Cobham, Paul A Dawson, Mark Harris, Elizabeth M Hurrion, David P Laplante, Brett M McDermott, H David McIntyre, Michael W O’Hara, Norbert Schmitz, Helen Stapleton, Sally K Tracy, Cathy Vaillancourt, Kelsey N Dancause, Sue Kruske, Nicole Reilly, Laura Shoo, Gabrielle Simcock, Anne-Marie Turcotte-Tremblay, Erin Yong Ping

**Affiliations:** Douglas Mental Health University Institute, Montreal, Canada; McGill University, Montreal, Canada; Mater Research Institute, Brisbane, Australia; The University of Queensland, Brisbane, Australia; University of New South Wales, Sydney, Australia; St. John of God Health Care, Brisbane, Australia; Mater Health Services, Brisbane, Australia; University of Iowa, Iowa City, USA; INRS – Institute Armand-Frappier (Université du Québec) and BioMed Research, Laval, Canada; Université du Québec à Montréal (UQAM), Montreal, Canada; Université of Montréal, Montreal, Canada; The University of Sydney, Sydney, Australia; The Royal Hospital for Women, Randwick, Australia

**Keywords:** Natural disaster, Pregnancy, Prenatal stress, Cortisol, Placenta, Fetal programming, Child development, Developmental origins of health and disease (DOHaD), DNA, Post-traumatic stress disorder

## Abstract

**Background:**

Retrospective studies suggest that maternal exposure to a severe stressor during pregnancy increases the fetus’ risk for a variety of disorders in adulthood. Animal studies testing the fetal programming hypothesis find that maternal glucocorticoids pass through the placenta and alter fetal brain development, particularly the hypothalamic-pituitary-adrenal axis. However, there are no prospective studies of pregnant women exposed to a sudden-onset independent stressor that elucidate the biopsychosocial mechanisms responsible for the wide variety of consequences of prenatal stress seen in human offspring. The aim of the *QF2011 Queensland Flood Study* is to fill this gap, and to test the buffering effects of Midwifery Group Practice, a form of continuity of maternity care.

**Methods/design:**

In January 2011 Queensland, Australia had its worst flooding in 30 years. Simultaneously, researchers in Brisbane were collecting psychosocial data on pregnant women for a randomized control trial (the M@NGO Trial) comparing Midwifery Group Practice to standard care. We invited these and other pregnant women to participate in a prospective, longitudinal study of the effects of prenatal maternal stress from the floods on maternal, perinatal and early childhood outcomes. Data collection included assessment of objective hardship and subjective distress from the floods at recruitment and again 12 months post-flood. Biological samples included maternal bloods at 36 weeks pregnancy, umbilical cord, cord blood, and placental tissues at birth. Questionnaires assessing maternal and child outcomes were sent to women at 6 weeks and 6 months postpartum. The protocol includes assessments at 16 months, 2½ and 4 years. Outcomes include maternal psychopathology, and the child’s cognitive, behavioral, motor and physical development. Additional biological samples include maternal and child DNA, as well as child testosterone, diurnal and reactive cortisol.

**Discussion:**

This prenatal stress study is the first of its kind, and will fill important gaps in the literature. Analyses will determine the extent to which flood exposure influences the maternal biological stress response which may then affect the maternal-placental-fetal axis at the biological, biochemical, and molecular levels, altering fetal development and influencing outcomes in the offspring. The role of Midwifery Group Practice in moderating effects of maternal stress will be tested.

**Electronic supplementary material:**

The online version of this article (doi:10.1186/s12884-015-0539-7) contains supplementary material, which is available to authorized users.

## Background

### Introduction

Retrospective epidemiological studies suggest that maternal exposure to a severe stressor during pregnancy (e.g., death of a close relative [1], foreign invasion [2]) increases the fetus’ risk for suboptimal growth, and for developing a variety of neurodevelopmental disorders later in life, such as autism [3] and schizophrenia [1,2,4]. The subsequent challenge is to uncover the process that is responsible for these effects: how much of the predictive power of prenatal maternal stress (PNMS) is due to the objective severity of the mother’s exposure to the event itself, how much is due to her subjective level of distress, how much to her hormonal response, and how might these aspects of the stress experience interact to alter risk for the unborn child? In addition, to what extent, and in what way, are the putative effects on the infant and child determined by the mother’s genetic profile, the child’s genetic profile, the child’s sex, and the gestational timing of the stressor?

When testing the fetal programming hypothesis prospectively, animal studies randomly assign pregnant animals to stress or non-stress conditions and find that maternal glucocorticoids (GCs) pass the placental barrier and alter fetal development, in particular influencing the function of the hypothalamic-pituitary-adrenal (HPA) axis and immune system [5-12], causing changes in a wide variety of physiological outcomes for the offspring. In animal studies, the type, severity, and timing of the prenatal stress can be varied and controlled. In humans, however, the gold standards of rigorous experimental methodology are difficult to meet because we cannot randomly assign pregnant women to different stress conditions to determine the effects on the fetus. Most human PNMS research has focused on women experiencing stressful life events. However, most of these events do not occur randomly and may be clustered with other confounding socioeconomic, genetic, and personality, or temperament traits. These potentially confounding variables may have an ongoing influence on the infant and child, independent of the stressful event. Therefore, without the rigor of a randomized, controlled research design, the internal validity of a study is called into question, and the relative contributions of the multitude of biopsychosocial mechanisms responsible for the wide variety of consequences seen in human offspring remain obscure. As long as mechanisms remain obscure, avenues for interventions to alter these consequences will also remain obscure.

In their seminal book, The Social Origins of Depression, Brown and Harris [13] described their rigorous method for determining whether life events that occur prior to women’s diagnosis for major depression may have been partly caused by the women’s developing psychopathology, or were clearly independent of the women’s own agency. Their Life Events and Difficulties Schedule has become the gold standard in social psychiatry research for assessing the severity and independence of life events in order to tease apart the putative causal mechanisms behind depression in women.

With respect to pregnant women and prenatal stress, a sudden, independent environmental event offers an opportunity to circumvent the obstacles to random assignment in human research on PNMS. Many forms of disaster distribute harm in quasi-random fashion, affect sizable communities that include large numbers of pregnant women, and have sudden onsets that allow the researcher to determine the moderating effects of the timing of the event in pregnancy.

In human research, it is also important to distinguish between the pregnant woman’s objective degree of exposure to an event and the intensity of her subjective distress in response to it. Although one may assume that two rats of the same strain will not differ appreciably in their level of distress at having their tails pinched, for example, the human stress response is highly individualized. Appraisal theory, which describes the intrapsychic processes that explain why two people experiencing the same event may have completely different emotional reactions to it [14-16], suggests that the objective degree of exposure to a stressor and the subsequent subjective distress are relatively independent of each other.

The goal of the QF2011 Queensland Flood Study is to capitalize on a natural disaster in order to expand current understanding of how a stressful experience during pregnancy impacts on a woman’s unborn child. Although we study elements of the stress experience from a natural disaster, we believe that our findings could be generalized to other forms of hardship experienced by pregnant women; as such, the Queensland floods serve as a general model of stress exposure in pregnancy, unencumbered by confounding variables, such as maternal genetic predisposition or temperament, that may self-select some women into stressful situations. We aim to determine the relative effects of several objective, subjective, cognitive, and hormonal components of the stress experience by tracking these aspects of stress as they cascade onto biological processes in mother, placenta, and fetus, and onto development through early childhood and beyond. In this article, we will summarize the background literature on PNMS, and describe the protocol for QF2011.

### The fetal programming hypothesis, DOHaD, and prenatal maternal stress

The fetal programming hypothesis [17-22], is often referred to as the Developmental Origins of Health and Disease (DOHaD). It stipulates that physical features and behaviors in offspring of prenatally stressed mothers result from permanent alterations in the structure and function of the offspring’s organs and systems, most likely through the impact of maternal cortisol on the fetus’ developing HPA axis [6]. Because a linear relationship exists between maternal and fetal cortisol levels, relatively small increases in maternal cortisol resulting from anxiety or stress can double fetal concentrations [23], resulting in significant structural changes such as smaller hippocampal volumes [24-27]. Furthermore, anxiety and/or stress in the mother not only increases her own circulating cortisol, it also reduces the activity of the cortisol barrier enzyme, 11β-hydroxysteroid dehydrogenasetype2 (11-βHSD2), in the placenta (which converts maternal cortisol into inactive cortisone before it passes to the fetus), elevating placental and fetal exposure to cortisol [24]. Increased maternal cortisol results in the dysregulation of HPA axis activity in the offspring [20, 21]. This link is important in that dysregulated HPA axis activity is implicated in psychopathological disorders in both adults and children, especially anxiety and depression [28]. The changes in fetal development induced by the maternal physical and emotional environment are not necessarily detrimental to the well-being of the fetus; indeed, some alterations may be adaptive by better preparing the fetus for the postnatal environment. However, high levels of PNMS exposure might have negative effects that override the fetus’ ability to adapt.

### Effect moderators

The association between PNMS and health outcomes may not be simple and linear, as a number of factors may moderate the effects of PNMS on the woman and her unborn child.

Maternal psychosocial variables may be found to moderate the effects of PNMS. For example, considerable literature debates the “social support buffering hypothesis” [29], the idea that effects of a life event will differ according to the degree of social support. There is also an extensive literature in psychology demonstrating that various styles of coping with a life event, emotion-focused versus problem-focused [30, 31], will impact on the subsequent distress experienced. For example, problem-focused coping tends to increase an individual’s self-efficacy and control, and to reduce anger, anxiety, stress, and physiological arousal [32, 33].

The timing of PNMS appears to be an important moderator of PNMS effects on the fetus. It may be less the *type* of disruption to fetal neural development and more the *timing* in utero that determines risk for negative outcomes [34]. Timing effects are a function of (a) the ontogeny of fetal development, (b) alterations in maternal biological and psychological reactivity to stress during pregnancy, and (c) fetal exposure to stress-related biological processes through placental function (e.g., production of CRH) and/or placental transfer of, for example, cortisol [35].

It is also feasible that both maternal and fetal genetics moderate the effects of PNMS on the HPA axis, especially following events that are novel and uncontrollable [36]. In the current project, we focus on the maternal-placental-fetal (MPF) axis because it serves as a prototypical system with well-defined physiological inter-relationships to specify how we analyze interactions between maternal and fetal genotypes, and the environment.

*In utero* stress exposure may have sex-specific outcomes [8, 37-41] with some studies suggesting greater vulnerability in males, and others in females, depending on the outcome measured. Also, in rodents and humans, the activity and sensitivity of placental 11-βHSD2 to stimuli appears to be sex-linked, with greater vulnerability in males, which may partly explain the sex specificity of some stress-induced phenotypes [42].

Finally, effects of PNMS on offspring may be moderated by the postnatal environment. Just as animal studies show that maternal behaviors can partially reverse adverse behavioral and cognitive outcomes in pups [43], similar findings are beginning to emerge in human studies [44, 45].

### Disaster research and Project Ice Storm

Our approach to PNMS research is to study pregnant women experiencing natural disasters. By their nature, disasters tend to have sudden onsets and are generally considered to be “independent stressors”. Using disasters as natural experiments in PNMS research presents an approximation of the randomization afforded by laboratory animal studies, and capitalizes on the relatively large potential subject pool following a disaster to a large community.

The first prospective study of prenatal stress from a natural disaster was Project Ice Storm [46] in Canada. Project Ice Storm (http://www.mcgill.ca/projetverglas/) was initiated soon after a series of ice storms struck Southern Québec in January 1998 resulting in power outage for more than 3 million people for as long as 6 weeks during the coldest months of the year, and causing 27 deaths. The Insurance Bureau of Canada and Environment Canada count the ice storm as the worst and most costly natural disaster in Canadian history. Project Ice Storm was designed to clarify the role of PNMS in a prospective longitudinal design. Women who had been pregnant during the ice storm, or who became pregnant within three months of the storm, completed questionnaires about their objective exposure to hardship because of the storm (e.g., levels of threat, loss, change, and scope), and about their degree of distress (post-traumatic stress-like symptoms) due to the storm; they also provided saliva samples to assess diurnal cortisol patterns. Project Ice Storm mothers and children are still being followed more than 15 years later.

Effects of PNMS from the ice storm have been found in nearly every domain of child development examined. Significant effects of the degree of maternal objective exposure to the disaster (but not maternal subjective distress) have been found on the children’s cognitive development [47-49], obesity [50], insulin secretion [51], brain development [52], and epigenetic signatures [53]. Effects of maternal subjective distress (and to a lesser extent, objective exposure) have been found on fingerprint asymmetry [54], autistic-like traits [55], and diagnoses of asthma [56] throughout childhood. Objective exposure and subjective distress from the ice storm have been found to interact such that the worst child outcomes are associated with mothers who presented a mismatch between objective exposure and subjective distress, that is, children of mothers with high objective stress but low subjective distress, or with low objective stress along with high distress [55]. There have also been significant timing effects: early pregnancy exposure seems to represent a window of vulnerability for cognition at age 2 years [47], and autism-like traits at age 6½ years [57], while second trimester exposure was associated with shorter gestation lengths and other birth outcomes [58], while worse motor outcomes have been associated with exposure in late pregnancy [59]. The sex of the child has often been found to moderate effects of PNMS: girls were found to be more vulnerable to effects of PNMS on asthma-related symptoms and diagnoses [56] and motor functioning [59], while boys seem to be more vulnerable for effects on fetal growth [58].

Although the Project Ice Storm sample is relatively small (n < 200), statistical power is enhanced by having a range of exposure levels from mild to severe; by the fact that the degree of exposure to the disaster was independent of family psychosocial attributes; by the acute onset of the event which allows exact dating of the stressor in the pregnancy; and by having detailed information on the women, their exposures, and their distress gathered relatively soon after the disaster. The independent nature of the disaster reduces the need to identify and control for all potential confounders, and allows one to disentangle the effects of the women’s objective exposure to the event from their cognitive appraisal of it, and from their subjective distress and hormonal responses.

Project Ice Storm has many limitations, however. It suffers from the fact that women were first recruited 5-6 months after the crisis such that, for half of the women, the reports of storm events were given only after birth. It is difficult to combine diurnal cortisol data collected from women who were still pregnant, and from others who had already given birth, at the time of sampling. Although Project Ice Storm has advanced the PNMS field, it requires replication with (1) another type of disaster, (2) speedier recruitment and baseline data collection, (3) data collected from the pregnant women before the disaster struck to be able to control for pre-disaster psychopathology, (4) assessments starting earlier in childhood and better long-term retention, (5) biological samples from mother, placenta, and fetus to test hypotheses about physiological mechanisms in PNMS and interactions, and (6) with an intervention to buffer the potential effects of PNMS.

### The 2011 Queensland floods, the M@NGO trial, and QF2011

The Australian state of Queensland experienced severe flooding, the worst in 30 years, in early-January 2011 affecting more than 200,000 people in more than 70 towns. Three quarters of Queensland was declared a disaster zone with more than $1 billion of reported damages: flooding reached its peak in the state capital of Brisbane on January 10th. Residents of 2,100 Brisbane streets were told to evacuate and 20,000 homes were inundated. Approximately 35-40 deaths have been attributed to the floods. Two studies have published results on the effects of these floods on the perceived physical and mental health of residents. Researchers using random-digit dialing telephone surveys of more than 6,000 residents between March and June of that year found that 62 % of respondents reported being affected by Queensland’s severe summer weather events “in any way”, including property damage (as much as 37 % for those in some suburbs), with 2 % living elsewhere at least temporarily [60]. Income was reduced for 17 % of respondents. Trauma impacts ranged from feeling “terrified, helpless or hopeless” (14 %) to thinking they might be “badly injured or die” (4 %). Up to five months after the summer disaster, 7 % of respondents reported being “still distressed” and 9 % were “worried about how they would manage”. Results from a self-report postal survey (n = 960) conducted in July 2011 indicated that having been directly impacted by the floods (i.e., residence or vehicles affected by flood waters) was associated with increased risk of worse overall (OR = 5.3) and worse respiratory (OR = 2.3) health, greater psychological distress (OR = 1.9), and problems sleeping (OR = 2.3), and with symptoms reflecting probable post-traumatic stress disorder (PTSD) (OR = 2.3) [61, 62]. As response rates from both studies were less than 50 %, results are not generalizable to the entire population. Nonetheless, these results suggest that the Queensland floods of 2011 had significant impacts on health, including distress in the general population; nothing is known from these studies about effects on pregnant women, however.

The QF2011 Queensland Flood Study was designed to address the shortcomings of Project Ice Storm and other PNMS research. QF2011 subjects were recruited either through a pre-existing clinical trial or, after the floods, directly into the study outside of the clinical trial.

The January 2011 floods occurred during the active recruitment period for a large, unrelated study of pregnant women. Since June 2010, the Mater Mothers' Hospital (MMH) in Brisbane had been involved in a multi-site randomized controlled trial (RCT) called the M@NGO trial (Midwives @ New Group practice Options (Trial registration: Australian New Zealand Clinical Trials Registry ACTRN12609000349246)) [63, 64]. This study pitted standard antenatal care against caseload midwifery care provided within a Midwifery Group Practice (MGP). Approximately 300 Brisbane women had already been recruited into M@NGO prior to the floods, and recruitment continued until the pre-determined end of M@NGO recruitment across all sites on May 31, 2011. For the full M@NGO trial, power analyses had determined that a sample size of 750 women was required to detect a difference, with 80 % power and a type 1 error of 5 %, in the primary, obstetric end-points for the M@NGO Trial; the secondary outcomes of the trial were determined through questionnaires offered at 6-12 weeks and 6 months postpartum [63]. Women were eligible for inclusion in M@NGO regardless of risk factors, and were recruited for the trial at less than 24 weeks of pregnancy, at which time psychosocial and demographic data were gathered.

Participants were randomly assigned to receive one of two forms of care: standard care (public hospital-based care or shared care with general practitioners) in which prenatal, labor, birth and postnatal care is provided by on duty staff – both midwives and doctors; or MGP which provides each woman with a primary midwife who coordinates and provides continuity through prenatal, labor, birth and postnatal domiciliary care (up to six weeks postpartum). The primary midwife is supported by her MGP colleagues (typically 3-4 midwives/MGP) who provide back up in the absence of the primary midwife. In this study, births occurred in-hospital with the primary midwife coordinating higher level care as needed, for women with identified risk factors. A key feature of this model is continuity in care provision with women having 24/7 access to their primary or back-up midwife [65, 66]. A 2013 Cochrane review of 13 trials (16,242 women) found midwife-led continuity of care was associated with benefits for mothers and babies, with no identified adverse effects compared with models of medical-led care including shared care [65, 66]. Benefits found included reductions in fetal loss prior to 24 weeks’ gestation, preterm birth, epidural anesthesia, episiotomies, and instrumentally assisted birth. Women’s chances of being cared for in labor by a known midwife, and having a spontaneous vaginal birth, were also increased in the midwifery-led continuity care model, as were the rates of maternal satisfaction [65, 66]. Expanding on the previous MGP literature described above, the M@NGO trial was the first to test continuity of care without excluding women at higher obstetric risk at trial entry; as such, the M@NGO participants represent a more real-world sample than in previous MGP research.

The ‘active ingredients’ of the MGP intervention are not completely understood [67, 68]. The 24/7 access to a known midwife, resuts in more personalized access to care if needed. Having a known midwife providing care in labor and the early postpartum period, and having postpartum support for several weeks if required may provide a buffer from psychosocial stress [66]. The power of MGP perinatal care to protect pregnant women and their fetuses against psychological stress (whether due to disasters, bereavement, or other hardships) is currently unknown; the subgroup from the M@NGO trial included in the QF2011 study may provide valuable data on the benefits of this model of care as an intervention to buffer against stress.

Following the Queensland flood, the QF2011 study was ‘piggy-backed’ onto the M@NGO trial. QF2011 recruited additional pregnant women to create a larger sample size, with M@NGO participants as an important embedded subgroup. The QF2011 recruitment questionnaires included assessments of maternal exposure to, and stress arising from, the floods. The QF2011 protocol included biological sampling from the birth and from the newborn, and added measures of maternal and infant outcomes at 6-12 weeks and 6 months postpartum to the M@NGO protocol, as well as re-administration of the flood-related assessments 12 months post-flood (January 2012). QF2011 also includes direct assessments of the mothers and their children at ages 16 months, 2½, and 4 years.

### Problem statement, objectives, hypotheses and research questions

Retrospective human studies suggest that maternal exposure to a natural disaster or other severe stressors increases the fetus’ risk for disorders in childhood and adulthood. Animal studies, human studies of life events or anxiety, and retrospective epidemiological studies all have a number of limitations that compromise the internal validity of the study design or its conclusions, and tell us little about the active ingredients of the prenatal stress effect.

The overall aim of this project is to replicate, expand, and improve upon findings from Project Ice Storm to increase understanding of the mechanisms responsible for the effects of maternal exposure to varying levels of hardship imposed by a severe, independent stressor: a natural disaster. We will determine the effects of various aspects of maternal stress due to the 2011 Queensland floods on the mother, her pregnancy and her infant, and the extent to which these effects may be moderated by other biological, psychological, and social factors. We will also determine whether MGP care can be shown to buffer pregnant women and their unborn children against any effects of the floods.

The primary research question that QF2011 will address is the following: **To what extent will the mothers’ objective exposure, cognitive appraisal, subjective distress, and cortisol levels from the 2011 Queensland floods explain variance in outcomes for the mother, the pregnancy, and the unborn child?** More specific hypotheses and research questions are presented below.

1. By what biological mechanisms do different components of the stress experience (e.g., objective, subjective) get translated through the Maternal-Placental-Fetal (MPF) axis to influence development of the unborn child?

Although much research on fetal-programming by PNMS is conducted on rodents, the functioning of the human placenta is distinct from that of rodents, and no appropriate animal model exists. To date, no human studies have been able to show the links between an objective environmental stressor, placental function and neurodevelopmental outcomes. It is also unknown how the cascade of stress through the MPF axis, emerging from objective stress, differs from those of subjective distress. To understand how PNMS affects the MPF axis, three hypothetical models will be studied:

**Model 1:***MPF neuroendocrine systems*. We propose that objective and subjective PNMS will activate a cascade of events leading to an increase in maternal, placental, and fetal cortisol terminating in altered HPA axis function and a lower stress response in the child. We predict that more severe objective/subjective PNMS will decrease activity of the placental/activity of the placental 11β-HSD2, resulting in increased levels of cortisol in placenta and fetal blood (cord blood). Moreover, more severe objective/subjective PNMS will also be associated with increased placental Corticotropin-releasing hormone (CRH) production that will, in turn, over-stimulate the maternal HPA axis and thus exacerbate maternal cortisol production and the inhibition of placental 11β-HSD2, resulting in higher levels of cortisol in placenta and fetal blood (cord blood).

**Model 2:***MPF immune systems and metabolic outcome*. We propose that more severe objective/subjective PNMS will be associated with increased maternal catecholamine levels (physiological response to stress) resulting in an abnormally high level of oxidative stress and pro-inflammatory cytokines in the placenta that will increase inflammatory and oxidative status of maternal and fetal blood, which will cause an increase risk in obstetric complications (such as preterm birth, low birth weight, intrauterine growth restriction and preeclampsia), which may then go on to predict future risk for metabolic and psychiatric difficulties in children later in life.

**Model 3:***Birth weight and body composition*: We propose that higher placental cortisol levels will cause a decrease in the production of placental hormones regulated by glucocorticoids (such as human placental lactogen (hPL), placental growth hormone (pGH), leptin, estrogens, progesterone, and glucose transporter type 1(GLUT1; lower maternal-fetal glucose exchange)) resulting in a lower placental efficiency (lower birth weight and higher placental weight), resulting in fetal growth restriction and/or altered programming of body composition in the child. We hypothesize also that maternal and fetal glucose, insulin, leptin, and other adipocytokines in blood will be involved in this process.

We also hypothesize that the effects observed in models 1-3 in placental function and in children will be more important for male fetuses compared to female fetuses.

(2) Midwifery Group Practice (MGP) care (in comparison with standard care) will protect pregnant women and their unborn children from the impact of objective levels of exposure and subjective levels of maternal distress arising from the 2011 Queensland floods.

Project Ice Storm found multiple effects of objective stress exposure on child outcomes*.* Although psychosocial treatments may reduce maternal anxiety and subjective stress after a disaster [69], they cannot alter the objective hardship and danger that was faced. Because QF2011 is piggy-backed onto the M@NGO study, we have a built-in RCT of prenatal care that commenced before the flooding that may demonstrate protective properties of this more personalized, and supportive, coordinated midwifery approach. We hypothesize that MGP care will buffer effects of flood stress (objective and/or subjective) on maternal outcomes, such as PTSD symptoms and depression, on perinatal outcomes (e.g., gestation length and birth weight), and on child outcomes.

(3) Maternal and child genotypes will moderate the effects of PNMS on outcomes. Some examples of such gene-by-environment interactions are listed here:
  - Maternal mental health:
    i. We hypothesize that maternal PTSD symptom severity (1 year post flood) may be explained by interactions between objective stress (especially Threat) and the maternal 5-HTTLPR (serotonin-transporter-linked polymorphic region) of the *SLC6A4* gene, controlling for potential confounders.
    ii. Postpartum (and continuing) maternal depression may involve interactions between objective stress (especially Loss) and the maternal 5HT2A gene (serotonin receptor 2A), controlling for potential confounders.
  - Child stress reactivity:
    i. The effects of PNMS on the child’s HPA axis will be moderated by infant genotypes associated with the stress response, such as CRH, CRHR1.
  - Epigenetics: Since Project Ice Storm has shown that maternal objective stress predicts DNA methylation in children at age 13 years [70], QF2011 will also endeavor to obtain biological samples throughout childhood for testing epigenetic hypotheses at a later date.

(4) Does maternal sensitivity mediate, or moderate, (or both) the effects of PNMS on child outcomes?

Research suggests that the post-natal environment may buffer the effects of prenatal maternal mood on child outcomes. Austin’s group found that maternal sensitivity moderated the impact of maternal antenatal anxiety on the child’s cognitive and behavioral outcomes at 7 months [45, 71]. There are no data, however, on whether maternal sensitivity can moderate the effects of an independent prenatal maternal stressor on child outcomes. By the same token, it is unknown whether an objective, independent stressor in humans will influence maternal behaviors, like sensitivity, which may then mediate effects of PNMS on the child. Thus, we hypothesize that objective exposure and subjective stress from the floods will predict HPA stress reactivity and cognitive development in QF2011 infants, and that this association may be mediated and/or moderated by maternal sensitivity.

(5) How do pre-existing levels of depression interact with peritraumatic responses to the Queensland floods to influence the trajectory of maternal mental health following exposure?

Because a measure of perinatal depression (the Edinburgh Postnatal Depression Scale (EPDS)) is administered routinely to all women in Australia at the time of hospital booking, levels of pre-existing maternal depression are available for our subgroup of QF2011 women who had already booked at the Mater hospital prior to the floods. Therefore, the QF2011 study is in a position to respond to unanswered questions about the natural history of maternal mental health in response to a disaster. We predict that depression before exposure to the disaster will interact with levels of distress and dissociation at the time of the flooding (i.e., ‘peritraumatic’) to explain variance in postpartum levels of depression, anxiety, PTSD-like symptoms, and positive mental health.

(6) If variance in physical characteristics, like fingerprint asymmetry and finger length ratios, are explained by levels of objective and subjective PNMS, what biological processes are they markers for?

King and Laplante have found that PNMS from the Québec ice storm explains significant variance in physical markers associated with neurodevelopmental disorders, such as dermatoglyphic (fingerprint) asymmetry [54], and the ratio of the lengths of the second and fourth fingers (2D:4D ratio) which is reputed to reflect *in utero* exposure to testosterone [72, 73]. But it is unknown by what process PNMS influences 2D:4D ratios and dermatoglyphic asymmetries. Once it is determined which biological variables in placenta and cord blood are sensitive to PNMS, we will determine which of these, if any, are then correlated with these neurodevelopmental markers in hands.

(7) To what extent do the severity of prenatal maternal objective/subjective stress, and/or maternal cortisol during pregnancy pre-flood, predict the HPA axis function of the infant/child, and what other outcomes are associated with altered HPA axis function?

Prenatal maternal stress research, particularly in animal models, has demonstrated the effects of PNMS on the development of the fetal HPA axis, which regulates the stress response (Glover et al., [74]; Charil et al., [5]). In humans, fetal exposure during early to mid-pregnancy, the period during which the HPA axis develops, seems particularly deleterious [75, 76]. Alterations in HPA axis have also been shown to be associated with a wide range of neurodevelopmental, psychiatric and metabolic outcomes [74, 77]. We aim to determine whether it is mere objective exposure to the stressor, or the maternal subjective distress, that accounts for altered HPA axis function in the infant/child, and which outcomes (e.g., attention, emotional difficulties, learning and memory recall, obesity) are associated with altered function.

## Methods/design: QF2011

### Participants

All women who agreed to participate in the study provided written informed consent. Inclusion and exclusion criteria differed slightly for M@NGO [63, 64] and for QF2011. Women were eligible for recruitment to the M@NGO trial if they were less than 24 weeks pregnant and were not planning caesarean section birth; QF2011 participants had to have been pregnant with a singleton pregnancy (at any gestation) and residing in the general vicinity of Brisbane on January 10, 2011. All participants were 18 years of age or older at recruitment, and were able to speak English fluently.

Recruitment for QF2011 began as soon as ethics approval was received (April 4, 2011), and continued to mid-January 2012 (12 months after the peak of the flood); as per original protocol, recruitment for M@NGO ceased on May 31, 2011 in both Sydney and Brisbane. Recruitment proceeded in 4 ways:

1. When new participants were recruited into M@NGO, they were also presented with the QF2011 protocol, and invited to participate in either or both studies. M@NGO recruitment at the Mater Hospital involved face-to-face, phone, and e-mail recruiting following a routine mail-out describing the study to all women attending the antenatal clinic.
2. The team contacted all existing M@NGO subjects who were eligible for QF2011 first by e-mail, then by cell phone (text message), then by telephone inviting them to also participate in the new flood study.
3. The team invited women who were pregnant and eligible for the QF2011 study, but not eligible for M@NGO, or who would have been eligible for M@NGO but were approached after M@NGO recruitment ceased, to participate in QF2011. A flyer inviting women to participate and to contact the recruitment team was included in the routine mail-out from the antenatal clinic. A researcher in the antenatal clinic actively recruited participants face-to-face on site and was available to speak to midwives and other potential participants about the study.
4. Finally, advertisements were placed in local newspapers, on radio, and on the internet for women who were pregnant at the time of the floods (even if they had already delivered).

As such, this study included 2 main groups of participants:

1. QF2011 women enrolled in M@NGO (n = 108, including 1 intrauterine death). These participants were enrolled in both M@NGO and QF2011. Some were already M@NGO study participants at the time of the floods and where subsequently recruited to QF2011; others were pregnant at the time of the floods and were recruited into both studies simultaneously. At recruitment to the QF2011 study, M@NGO women may still have been pregnant or they may have already given birth as the inclusion criteria stated only that they must have been pregnant on January 10, 2011.
2. QF2011 women not enrolled in M@NGO (n = 122). This group included women who had declined participation, did not meet the selection criteria for the M@NGO study, or who were recruited after the close of M@NGO (May 31, 2011).

### Instruments

See Table 1 for the M@NGO and QF2011 data collection tools.

**Table 1:** **M@NGO (M) and QF2011 (Q) data collection tools**

	**Pregnancy timeline**								**Flood timeline**	
	**M@NGO recruit (12 – 14) wk pregnancy** ^**1**^	**M@NGO 36 wk pregnancy**	**At/after delivery**	**6-12 wks**	**6-mos**	**16-mos**	**2 ½ yr**	**4 yr**	**Flood assessment @ recruitment in QF**	**12 mos post- flood**
**Maternal assessment: Questionnaire**										
Lifestyle choices (smoking, drinking, exercise)		M			M					
Relationship Questionnaire		M							Q	
Edinburgh (EPDS)	MATRIX			M	M					
Subjective Health				M	M					
Baby’s Health				M	M					
Feeding History				M	M					
Satisfaction with hospital care				M						
Satisfaction with care in labour & birth				M						
Experience of labour and birth				M						
About the pregnancy				M						
Health and Wellbeing Questionnaire (sf-36)					M					
Experience of Motherhood Questionnaire (EMQ)					M					
Demographics (different versions used)		M				Q	Q	Q		
Parenting Stress Index: short form (PSI)					Q	Q	Q			
Social Support Questionnaire						Q	Q	Q		
Brief Cope (including Brief R-Cope)						Q	Q	Q	Q	Q
Life Events Scale (LES)					Q	Q	Q	Q		
Parent’s Handedness (collected once)						Q	Q	Q		
Depression, Anxiety and Stress Scale-DASS						Q	Q	Q		
Centre for Epidemiologic Studies - Depression (CES-D)							Q			
Objective Stress: QFOSS									Q	Q
IESR (PTSD symptoms - subjective stress)									Q	Q
State Trait Anxiety (STAI)				Q	Q		Q		Q	Q
Peritraumatic Distress Inventory (PDI)									Q	Q
Peritraumatic Dissociation (PDEQ)									Q	Q
Positive Mental Health Continuum-Short									Q	Q
Pregnancy questions (cold & flu)				Q						
Childhood Trauma Questionnaire (CTQ)										Q
Adult Trauma History Questionnaire (THQ)										Q
Theory of Mind							Q			
Parenting and Family Adjustment								Q		
Relationship Quality								Q		
**Maternal assessment: Face-to-face evaluation or phone Interview**										
Breast Milk Consumption Interview						Q				
Maternal Sensitivity (Emotional Availability Scales)						Q	Q			
MINI diagnostic interview (conducted once)						Q				
Mother height, weight, and % body fat (father if present)						Q	Q	Q		
Wechsler Adult Intelligence Scale (WAIS)										
National Adult Reading Test (NART)								Q		
**Child assessment: Maternal questionnaire**										
Ages & Stages Questionnaire (ASQ)				Q	Q	Q	Q	Q		
MacArthur-Bates Communicative Development Inventory-III						Q	Q			
Short Temperament (STSI or STST or STSC)				Q	Q	Q	Q	Q		
Brief Infant-Toddler Social and Emotional Assessment (BITSEA)						Q				
Child Behavior Checklist (CBCL)							Q	Q		
Autism Spectrum Rating Scales (ASRS)							Q	Q		
Child’s Handedness						Q	Q	Q		
Breastfeeding Questions									Q	Q
Asthma and Allergies Questionnaire								Q		
Child Health Status								Q		
Preschool Anxiety Scale								Q		
**Child assessment: Teacher questionnaire**										
Caregiver-Teacher Report Form (C-TRF)								Q		
**Child assessment: Face-to-face**										
Bayley Scales of Infant and Toddler Development						Q	Q			
Solo Free Play protocol						Q	Q			
Wechsler Preschool Primary Intelligence Scale (WPPSI)								Q		
Beery-Buktenica Visual-Motor Integration (VMI)							Q	Q		
Attention and executive function (NEPSY)								Q		
Peabody Picture Vocabulary Test (PPVT)								Q		
Maternal Separation Stressor (up to 3 mins)						Q	Q			
Behavioural measure of stress reactivity						Q	Q			
Anthropometric measures						Q	Q	Q		
Height and weight						Q	Q	Q		
Body composition								Q		
Theory of Mind							Q	Q		
Effortful Control								Q		
Parental Involvement Task								Q		
**Medical records**										
Labour/Delivery; Skin-to-skin time, Birth weight; Gestational age; APGAR; Length; Head circumference; Neurological @ Dch; Maternal height and weight; BMI; Weight gain			M							
Baby sex; Anomalies; Ultrasound report; Blood test reports; Glucose tolerance; Cord blood gas report, Doppler report of uterine and umbilical blood flow, Rx in pregnancy			Q							
**Baby biological samples**										
Placenta sample (inc. weight, size, etc)			Q							
Cord blood			Q							
Umbilical cord			Q							
Cord length			Q							
Cortisol response to stressful situation (2 samples = 0 + 20 min)						Q	Q			
Diurnal cortisol (6 samples for 2 days)						Q	Q			
Testosterone (assayed from waking saliva)						Q	Q			
Child DNA (Saliva kits)							Q			
**Maternal biological samples**										
Diurnal cortisol in mother: 10 samples 2 days									Q	Q
Maternal blood sample at 36 weeks		Q							Q	
Maternal DNA (saliva)			Q			Q	Q			

Note. M = M@NGO protocol; Q = QF2011 protocol; ^1^QF2011 ONLY women only received one MANGO questionnaire before birth.

### Instruments in the M@NGO protocol

A detailed presentation of the M@NGO protocol is provided in the 2011 protocol publication [63].

#### *36 Weeks pregnancy*

At 36 weeks, a demographics questionnaire, lifestyle questionnaire (smoking, alcohol consumption, etc.), and relationship questionnaire were administered.

#### 6-12 weeks and 6 months postpartum

At 6-12 weeks postpartum, satisfaction with hospital care during labor and birth, experience of labor and birth, and information about the pregnancy in general were assessed. At both 6-week and 6-month assessments, questionnaires which included the Edinburgh Postpartum Depression Scale (EPDS; [78]) were distributed to all study participants. [78, 79]. As part of our duty of care, our policy was that women who scored 12 or greater on the EPDS, or who indicated intended self-harm, were followed up with a telephone call from a research midwife, and referred to appropriate services as needed. The questionnaires at 6 weeks and 6 months postpartum also included scales of subjective health, infant health, and feeding history.

#### Routine database hospital chart review

After birth, M@NGO researchers extracted routinely-collected data from hospital databases such as antenatal care and complications, mode of birth, birth weight, gestational age at birth, APGAR scores, maternal and newborn complications, skin-to-skin time, birth length, head circumference, breastfeeding commencement and admission to neonatal intensive care nursery. Additional data were obtained from a medical record audit (e.g., number of care providers in labor).

### Instruments in the QF2011 protocol

Women recruited into QF2011 were administered the same questionnaires, and had their medical information gathered, as described in the M@NGO protocol above. QF2011 added several questionnaires and procedures to the M@NGO protocol at recruitment, 36 weeks pregnancy, and at the 6-12 weeks and 6 months postpartum assessments. The QF2011 protocol also included a repeat of the flood-related questionnaires at 12 months post-flood (January 2012). In addition, QF2011 added procedures conducted at the birth, at the face-to-face assessments when the infants were 16 months and 2½ years of age, and will be conducting further assessments at age 4 years.

#### Flood-related questionnaires

##### Objective flood stress exposure (recruitment and 12 months post flood)

Disasters are characterized by “disruption exceeding the adjustment capacity of the affected community” [80]. Important dimensions of disasters include loss (of persons or property), threat to life or physical integrity, scope (proportion of the community affected), blame for the event (man v. nature), familiarity (experience with similar events), speed of onset, duration, amount of displacement, and potential for recurrence [81-86]. Because events differ along so many dimensions, disaster questionnaires must be tailor-made. For Project Ice Storm, we formulated objective questions that would reflect the participants’ experiences related to four of these categories of exposure: Threat, Loss, Scope, and Change. Each dimension was scored on a scale of 0–8, ranging from no exposure to high exposure. A total objective stress score was calculated by summing scores from all four dimensions; because there was no theoretical basis to believe that any one of the four dimensions of our scale would be more predictive than the other dimensions, and based on McFarlane’s study of Australian fire fighters [87], each dimension was weighted equally to obtain the total score [88]. The final scale was called STORM32.

In 2008, we modified the STORM32 scale to assess objective stress exposure for a new project: The Iowa Flood Study [89]. By taking into account experiences unique to flooding, and removing ice storm-related items, we created the Iowa Flood 100 (IF100) scale which included the same four domains as STORM32 (Threat, Loss, Scope and Change) with a possible 25 points per domain.

For QF2011, we added items to each domain of the IF100 questionnaire to more fully represent the women’s flood-related experiences, and allotted a maximum of 50 points per domain, resulting in the QFOSS (Queensland Flood Objective Stress Scale) with a maximum possible score of 200 points (Additional file 1). This scale was administered to all women at recruitment. It was re-administered at 12 months post-flood (January 2012) to update the values of financial loss, damage to house and property, experiences dealing with insurance companies, etc. Although recruitment and 12 month QFOSS scores are kept separate for some analyses, most analyses will be conducted with a final QFOSS score that integrates information from both. Because the IF100 is embedded within the QFOSS, a Queensland IF100 (QIF100) score can also be obtained for direct comparison with the Iowa study.

##### Cognitive appraisal (recruitment and 12 months post flood)

To assess the mothers’ cognitive appraisal of the flood, we included the following item in the questionnaire packages about flood exposure and distress: “Overall, what were the consequences of the flood on you and your family?”; response options were on a five-point scale of “Very negative” (1), “Negative” (2), “Neutral” (3), "Positive" (4), and “Very positive” (5).

##### Peritraumatic distress and dissociation (recruitment and 12 months post flood)

Peritraumatic measures ask the participant to recall how they *had felt* at the time of the disaster. We used the 13-item Peritraumatic Distress Inventory (PDI; [90]) and the 10-item Peritraumatic Dissociative Experiences Questionnaire (PDEQ; [91]). These measures allow the researcher to quantify the DSM-IV “trauma exposure” criterion for a PTSD diagnosis. These scales have been used in more than 200 studies around the world, including studies of 9/11, and traumatic childbirth [92].

##### Subjective, enduring distress from the flood (recruitment and 12 months post flood)

The severity of PTSD symptoms was assessed using the 22-item Impact of Event Scale – Revised (IES-R; [93]). While the PDI and PDEQ ask the respondent to recall their feelings at the time of an event, the IES-R concerns the respondent’s current and ongoing symptoms due to the event. The IES-R yields a total score as well as scores for three categories of PTSD symptoms: intrusive thoughts, avoidance, and hyperarousal. Participants respond on a 5-point Likert scale, from “not at all” to “extremely”, the extent to which each behavior describes how they felt in relation to the floods during the preceding 7 days. The IES-R was chosen because it is widely used and will allow us to compare the data from QF2011 with those from Project Ice Storm and from The Iowa Flood Study. Although the IES-R is not a diagnostic instrument *per se*, it can be used to screen for cases who may meet diagnostic criteria for PTSD. Published suggestions for cut-off scores to screen for potential PTSD with the IES-R include scores of 22 [94], 25 [95], and 33 [96].

A number of women were recruited into QF2011 more than 10 months after the floods (n = ~50). Given that a 12-month post-flood questionnaire was planned, which repeated the administration of the recruitment questionnaire, the steering committee chose to send these women a single questionnaire at 12 months post flood rather than have them repeat the same questionnaire 2 months apart. As such, these women lacked a “recruitment IES-R” score. In order to impute these missing values, multiple regression was used to estimate their recruitment IES-R scores from post flood questionnaire responses. We used the dataset of women who had completed both the recruitment and the 12-month post flood questionnaires, and created separate algorithms for each IES-R sub-scale (Intrusions, Avoidance and Hyperarousal). Using this set of complete data, we regressed each recruitment IES-R sub-scale score on 12-month post-flood IES-R sub-scale scores, and other related scores that maximized the variance explained (STAI, PDI, PDEQ, MHC, etc.). The models explained 35.5 % (Avoidance), 44.2 % (Intrusions), and 49.1 % (Hyperarousal) of the variance in the recruitment scores. The regression coefficients from these results were used to create the equations we used for imputing the missing IES-R sub-scale scores for women who had not completed a Recruitment questionnaire. Once the sub-scales had been successfully imputed, IES-R Total scores were computed as usual by summing the sub-scales.

Maternal psychological health, psychosocial factors and other maternal measures

*Maternal anxiety (recruitment, 12 months post-flood, 6-12 weeks, 6 months, and 2½ years)*

The State-Trait Anxiety Inventory (STAI; [97]) is a valid and reliable self-report measure of how one generally feels (trait) or currently feels (state). We administered the State scale of the STAI at recruitment, 12 months post flood, and at 6-12 weeks and 6 months. At 2½ years we administered the Trait scale of the STAI. Each scale has 20 items and participants are asked to rate statements (e.g., “I am happy” and “I lack self-confidence”) on a 4-point Likert scale (rated from Almost Never to Almost Always).

*Depression (6-12 weeks and 6 months postpartum)*

As noted in the M@NGO protocol section, the EPDS (Edinburgh Postpartum Depression Scale) was administered as part of M@NGO to assess maternal depression in pregnancy and again at 6-12 weeks and 6 months postpartum.

*Depression, Anxiety and Stress Scales (16 months, 2½ and 4 years)*.

For maternal mental health at later assessments, we administered the 21-item short form of the Depression Anxiety Stress Scales (DASS-21 [98-100]) which has three independent scales (i.e., anxiety, stress, and depression) and has been used widely in perinatal samples.

*Maternal depression (2½ years)*

Maternal depression was also assessed with the Centre for Epidemiologic Studies Depression Scale *(*CES-D; [101]) screening questionnaire. This is commonly used to assess feelings of depression in the general population. Women rated 20 statements (e.g., “I felt that I was just as good as other people” and “I felt depressed”) for how they felt during the past week on a 4-point scale ranging from “Rarely or none of the time (<1 day)” to “Most or all of the time (5-7 days).”

*Positive mental health (recruitment and 12 months post-flood)*

Positive mental health was assessed with the 14-item Mental Health Continuum – Short Form (MHC-SF [102]), which provides scores on Social, Emotional, and Psychological Well-Being. This scale has excellent psychometric properties. Extreme scores on this scale can define subgroups of “flourishing” and “languishing”.

*Coping style (recruitment, 12 months post-flood, 16 months, 2½ years, and 4 years)*

The Brief COPE includes 28 items: two items for each for the 14 coping strategies [103]. The Brief R-COPE assesses the frequency of 14 religious coping strategies which load onto positive and negative religious coping factors [104].

*Prior trauma (12 months post-flood)*

Because we have found that childhood trauma is associated with lower diurnal cortisol values in adulthood [105], women were asked to complete the Childhood Trauma Questionnaire (CTQ; [106, 107]), and the 24-item Trauma History Questionnaire (THQ; [108]) when the second set of salivary cortisol samples was taken. The CTQ provides information concerning instances of emotional, sexual, and/or physical abuse and emotional and physical neglect occurring before age 18 years. The THQ assesses whether individuals have experienced traumatic events such as crime, disasters, and physical or sexual assault after the age of 18.

*Life events (6 months and 16 months, 2½ years, and 4 years)*

Because for some women, the flooding may have been the least of their problems during the pregnancy, or the floods may have compounded a host of other events, we need to control for this potential confound. The Life Experience Survey (LES; [109]) lists 57 life changes, such as death of a spouse or a promotion at work; we reduced the number of items to the 29 more common occurrences. Respondents first indicate whether the event occurred or not, and then rate the impact of the event (if it occurred) on a 7-point Likert scale ranging from "Extremely Negative" to "Extremely Positive" and are asked to indicate the month and year of any events. Women indicate events that occurred in the preceding 24, 25, 12, and 18 months, respectively at the 6 month postpartum, 16 months, 2½ years and (forthcoming) 4 year assessments.

##### Social support (16 months, 2½ years, and 4 years)

The six-item Social Support Questionnaire (Short Form) (SSQSF; [110]) seeks not only information about the support available to mothers in various situations, but also their level of satisfaction with the support available. This is crucial as perceptions of social support may be just as important as the actual receipt of same [111]. Part 1 asks the respondent to list all of the people who fit the description of the question, while part 2 asks how satisfied they are, in general, with the support provided by these people. The SSQSF has high internal reliability and correlates highly with the longer version of this tool [110].

##### Parenting stress (6 and 16 months, and 2½ years)

The Parenting Stress Index: Short Form (PSI; [112, 113]) contains 36 items with statements regarding parenthood (e.g., “my child smiles at me less than I expected”) for which mothers report their level of agreement on a 5-point scale from “strongly agree” to “strongly disagree”.

##### Parental handedness (16 months)

Because left or mixed handedness in the child may reflect neurodevelopmental insult [114, 115], we also assessed parental handedness as a control variable. In the Parental Handedness Questionnaire [116] mothers were asked to indicate which hand they use for a list of 11 activities, including writing, drawing, and throwing a ball. They were also asked to indicate if they knew which hand the child’s biological father used for the same activities.

##### Maternal Theory of Mind (TOM) (2½ years)

TOM refers to the understanding that other people have mental states (e.g., beliefs, understanding, intentions, desires, and emotions) that may differ from one’s own. Poor TOM is a common trait of autism. To control for parental TOM when examining associations between PNMS and TOM in the children, parental TOM will be assessed using the Empathy Quotient (EQ [117]). The EQ is a list of 60 statements about how easily the participant understands other people's feelings and how strongly they are affected by other people's feelings (e.g., “I often find it difficult to judge if something is rude or polite"). The statements are rated on a 4-point scale from ‘strongly agree’ to ‘strongly disagree’.

##### Partner Relationship (4 years)

The Relationship Quality Index is a 6-item scale that asks respondents to rate on a 7-point Likert scale, their level of satisfaction in various areas of their relationship (i.e. stability, strength, etc.). Response options range from 1 “very strongly disagree” to 7 “very strongly agree” for the first five items. Item six relates to the overall level of happiness in the relationship, and is scored from 1 “unhappy” to 10 “perfectly happy” [118]. The overall score is computed by taking the sum of all six items, with higher scores representing greater satisfaction within the partner relationship.

##### Parenting and family adjustment (4 years)

The 40-item self-report Parenting and Family Adjustment Scale (PAFAS) assesses parent and family adjustment, as well as parenting practices. The Parenting Scale is comprised of two domains: parenting practices (17 items) and parent-child relationship (11 items); while the Family Adjustment Scale is comprised of three domains: parental emotional maladjustment (5 items), family relationships (4 items), and parental teamwork (3 items) [119]. Respondents are instructed to rate each statement on a scale from 0 “not at all” to 3 “very much (most of the time)”, with respect to the past four weeks. The measure is scored by taking the sum of all items, some of which are reverse coded. The Family Adjustment Scale has been found to have good internal consistency and satisfactory construct and predictive validity [120].

#### Maternal-rated scales of child development

*Infant temperament (6-12 weeks, 6 months, 16 months, 2½ years, and 4 years)*

The Short Temperament Scale for Infants (STSI) was developed as part of the ‘Australian Temperament Project: A Series of Studies of Australian Temperament, Development and Behavior in Australian Children’ [121]. The STSI is a 30-item questionnaire in which parents rate the occurrence of common infant behaviors on a 6-point scale ranging from 1 (almost never) to 6 (almost always). The items yield five scales which measure the temperament dimensions of approach-withdrawal, rhythmicity, cooperation-manageability, activity-reactivity, and irritability. An overall “easy/difficult” score is calculated as the mean of the approach-avoidance, cooperation-manageability, and irritability scales. Infants scoring one standard deviation above and below the normative mean are classified as “difficult” and “easy” respectively. The STSI was administered to mothers at 6-12 weeks and 6 months. The companion Short Temperament Scale for Toddlers (STST; [122]) was administered at 16 months and 2½ years, and we will administer the version for older children, the Short Temperament Scale for Children (STSC; [122]), at 4 years.

##### Developmental milestones (6-12 weeks, 6 months, 16 months, 2½ years, and 4 years)

Mothers have been completing the appropriate age-range versions of the Ages and Stages Questionnaire (ASQ-3; [123]) at every assessment: at 6-12 weeks postpartum, the 2-month version was used; at every other age, precise, age-appropriate versions were administered. The ASQ-3 is a 30-item parent-rated questionnaire that assesses five domains of development: communication, gross motor, fine motor, problem solving, and personal-social. Parents complete the questionnaire by answering each item using a 3-point Likert scale: 'Yes', 'Sometimes', or 'Not Yet'.

*Language abilities (16 months and 2½ years)*

Language development was assessed using the short form of the MacArthur-Bates Communicative Development Inventory: Words and Sentences (MCDI; [124]) at 16 months. The mother was given a list of 100 words and asked to indicate whether her child “understands” (receptive vocabulary) or “says” (productive vocabulary) each word. At 2*½* years an age-appropriate version of the MCDI-III was used that contains 100 words to assess the child’s productive vocabulary; the scale asks additional questions about how the child combines words into sentences and how they use language to ask questions, and about mean utterance length. This maternal report helps to avoid situational and temperamental factors that can affect children’s performance at face-to-face assessments, such as lack of interest or cooperation in the tasks, perhaps due to illness and/or anxiety [125].

*Infant handedness (16 months, 2½ years, and 4 years)*

Following procedures described by Glover et al. [116] we ask mothers which hand their child usually uses for the following five activities: drawing or coloring, throwing a ball, hitting things, stacking blocks, and using a spoon.

*Social-emotional development (16 months)*

The Brief Infant-Toddler Social Emotional Assessment (BITSEA; [126]) is a 42-item questionnaire that aims to identify children at risk for, or currently experiencing, social-emotional/behavioral problems and/or delays in social-emotional competence. It addresses four domains: Externalizing, Internalizing, Dysregulation, and Competence. This tool allows mothers to provide information about how their children behave in different situations.

##### Childhood Anxiety (4 years)

The 34-item Spence Childhood Anxiety Scale (SCAS) scale for preschoolers [127] provides information on children’s anxiety-like symptoms. For the first 28-items, parents report on the frequency at which an item is true for their child: 1 (not at all) to 5 (very often true). Item 29 is an open-ended question about whether their child has experienced a traumatic event. Items 30-35 relate to whether the child has exhibited post-traumatic symptoms following the traumatic event, and are not scored. The 28 items provide an overall measure of anxiety, as well as data on six childhood anxiety sub-scales: generalized anxiety, social anxiety, obsessive compulsive disorder, physical injury fear, and separation anxiety.

##### Autism spectrum traits (2½ and 4 years)

The 15-item Autism Spectrum Rating Scales (ASRS; [128]) was designed to effectively identify symptoms, behaviors, and associated features of Autism Spectrum Disorders (ASD) in children and adolescents aged 2 to 18 years. Using a 5-point scale mothers indicate how often they observe their child engaging in specific behaviors. Sub-scale scores for Social/Communication, Unusual Behaviors, Peer Socialization, Adult Socialization, Atypical language, and Stereotypy can be calculated.

##### Asthma and allergies (4 years)

In order to investigate the role of PNMS in influencing the children’s developing immune system, mothers will complete a 16-item questionnaire pertaining to their own symptoms of asthma, eczema and allergies, and those of their child and partner. The questionnaire was adapted from the International Study of Asthma and Allergies in Childhood (ISAAC) questionnaire [129, 130].

##### Child’s health status (4 years)

Information on the child’s health over the past two years will be assessed using a 9-item questionnaire completed by the mother. For each question (e.g., Did your child suffer from a concussion or head injury/experience a loss of consciousness/experience a high fever with delirium or coma, etc.), mothers will be asked to answer “yes” or “no”, and if “yes”, report on the child’s age at the time of the event, and if possible further details describing the situation.

##### Internalizing and externalizing problems (2½ and 4 years)

Internalizing and externalizing behavior problems are assessed by maternal report using the Child Behavior Checklist (CBCL; [131]), which is the most frequently cited measure of child psychopathology and has psychometric properties suitable for research. The CBCL consists of 100 statements about the child's behavior (e.g., Acts too young for his/her age). Responses for each item are 0 = Not True, 1 = Somewhat or Sometimes True, to 2 = Very True or Often True. The Internalizing Problems scale combines the Emotionally Reactive, Anxious/Depressed, Somatic Complaints, and Withdrawn sub-scales. The Externalizing Problems scale combines the Attention Problems and Aggressive Behavior sub-scales. A Sleep Problems sub-scale can also be obtained. This questionnaire replaces the BITSEA, administered at 16 months.

#### Teacher questionnaire

##### Internalizing and Externalizing Problems (4 years)

In order to have a third-party assessment of the child’s typical behavior, the children’s preschool teacher or day-care provider will rate internalizing and externalizing behavior problems using the Caregiver-Teacher Report Form (C-TRF). This is the teacher version of the Child Behavior Checklist (CBCL) described above [131], and has identical items and scales.

#### Telephone interviews

##### Breast milk consumption (Recruitment, 12-month post-flood, 16 months and 2½ years)

We have collected data on maternal breastfeeding history since the infant’s birth to estimate the total quantity of breast milk intake. The recruitment questionnaire and 12-month post-flood questionnaires included items asking mothers if they had breastfed their babies, for how long, and if they were currently breastfeeding. We conducted a telephone interview at 16 months and again at 2½ years if breastfeeding went beyond 16 months. Mothers were asked how long they practiced exclusive, predominant and mixed breastfeeding, respectively. The phone interview took up to 10 minutes in mothers who had breastfed. These data will allow greater understanding of any interactions between maternal stress and breast milk intake, and influence on infant development.

##### Maternal psychiatric diagnosis (16 months)

Mothers completed the semi-structured Mini-International Neuropsychiatric Interview-Plus version 6 (MINI-Plus 2010; [132]) during a phone interview with a clinical psychologist to determine any current or lifetime psychopathology diagnoses. We obtained information about the timing of any identified psychiatric episodes in relation to the participant’s pregnancy and the event of the 2011 Queensland flood.

#### Face-to-face assessments: mother and child (16 month, 2½ years, and 4 years)

##### Maternal height, weight and body composition (16 months, 2½ and 4 years)

These measures will be used as covariates in analyses of child physical outcomes. Maternal height and weight were obtained during the 16-month and 2½-year assessments. In addition, percent body fat (using Tanita electronic scales) (www.tanita.com) was assessed at 2½. All measures will be repeated at 4 years.

##### Maternal general intelligence (4 years)

Maternal IQ will be estimated using the National Adult Reading Test (NART; [133]). The NART contains a list of 50 increasingly difficult words that are all irregular to the common rules of pronunciation. Individuals are asked to read the words aloud and the number of errors in pronunciation is recorded. Predicted Full-scale IQ and Verbal or Performance IQ is estimated by entering the number of errors into one of three formulas (e.g. Predicted Full-scale IQ: 130.6 – (1.24 X Errors). The NART has high inter-rater (0.97) and test-retest (0.98) reliabilities [134, 135]. It has also been demonstrated that the NART loads highly (0.85) on the same general intelligence factor that is obtained when factor analysis is performed on the sub-scales of the Wechsler Adult Intelligence Scale [136].

##### Child body composition measures (16 months, 2½ and 4 years)

Children’s height, weight and head circumference were measured, along with anthropometric measures, at 16 months and 2½ years, and will be repeated at 4 years. Anthropometric measures include mid-upper arm, waist and calf circumferences, as well as triceps and subscapular skinfolds. These measures allow calculation of body composition, which provide a better indication of metabolic risk than weight alone [137].

##### Cognitive and motor functioning (16 months and 2½ years)

The Bayley-III Scales of Infant and Toddler Development [138] were used to measure cognitive and motor development. The cognitive scale includes items that assess thinking and problem solving. The motor scale assesses fine and gross motor skills. The Bayley provides four types of norm-referenced scores: scaled scores, composite scores, percentile ranks and growth scores. It has been used extensively in research and is generally recognized as the ‘gold standard’ tool to assess development of very young children.

##### IQ (4 years)

The Wechsler Preschool Primary Intelligence Scale – Australian Standardized Version-III (WPPSI-III Australian; [139]) -- will be administered to assess the children’s intellectual functioning in general, verbal, performance, and cognitive domains. It is considered the gold-standard IQ test in early childhood. The Block Design and Information sub-scales will be administered as this dyad provides an excellent estimation of the Full Scale IQ (r = 0.92).

##### Visual-motor integration (2½ years)

The Beery-Buktenika Developmental Test of Visual Motor Integration – 6^th^ edition (VMI; [140]) was used to test children’s perceptual-motor ability. The child was presented with a booklet with a drawing on one page and a blank page opposite on which the child was to reproduce the image. The drawings are rated for accuracy and scored according to age norms.

##### Behavioral stress reactivity (16 months and 2½ years)

We included a brief stressor in the face-to-face assessments in order to evaluate the child’s stress reactivity, both biologically (see below) and behaviorally. At both ages, the mother left the child alone in the testing room for up to three minutes while the child was observed via one-way mirror. The separation was terminated prior to three minutes if the child demonstrated excessive crying, or at the mother’s request. All separation sessions were videotaped for later behavioral coding. The separation-reunion clip will be coded for infant stress reactivity using an in-house coding scheme currently in development.

##### Theory of Mind (2½ and 4 years)

TOM permits young children to understand their social world and predict and make sense of other people’s behavior. For a child to exhibit TOM, they must disengage thinking about themselves and understand that other people may have mental states that differ from their own [141]. We used two Diverse Desires tasks to assess TOM at 2½ years of age. In the first task, the child is presented with a choice of two snacks (apple and cookie) and asked which he/she prefers. The child is then told that Big Bird prefers the other snack. Then the child is asked which snack Big Bird would choose to eat, given the choice. The second task is identical, but involves two play activities (bike riding or a slide) and Snoopy. The child receives one point for each task for which they correctly identify the “other desire” rather than their “own desire” in answer to the question.

At 4 years, we plan to assess TOM using the Unexpected Contents Task [142] which, similar to the Diverse Desires tasks, tests the child’s ability to make a judgment about what other characters know and what they are likely to do. The child is shown what is inside a distinctive container (e.g., pencils in a Smarties box) and has to judge what another character, who did not see what was in the box, believes to be inside the container.

##### Attention and executive functioning (4 years)

Attention and Executive Functioning will be assessed using the Statue sub-scale of the Developmental NEuroPSYchological Assessment – second edition (NEPSY-II; [143]). For this task, the child is asked to remain still with their eyes closed for 75 seconds and inhibit impulses to respond to distracting sounds. The number of instances in which the child does not remain still is recorded.

##### Effortful control (4 years)

Effortful control describes the ability to exert self-control with the intention of obtaining greater delayed reward(s). An adaptation of the ‘marshmallow’ self-control task developed by Mischel, Ebbesen and Zeiss [144] will be used to assess children’s level of effortful control. Children will be instructed to sit in a room, alone at a table, with a desired snack in front of them. They will then be given the choice to either eat the desired snack, or wait ten minutes to receive two of their desired snacks. The time before the child eats their snack(s), and the child’s behavior throughout the task, will be recorded for later analysis.

##### Language development (4 years)

The Peabody Picture Vocabulary Test, 4^th^ Edition (PPVT-4; [145]) will be used to assess receptive vocabulary (comprehension). The researcher presents the child with a book containing four pictures per page and asks the child to point to the picture that best represents the vocabulary word. The total number of correctly identified pictures can be converted to a standard score, percentile rank, or mental age.

#### Video recordings and ratings

##### Play levels (16 months and 2½ years)

Children’s play quality is an indirect measure of cognitive development, unencumbered by shyness or other social behaviors that may inhibit children from achieving their full potential when assessed by an examiner. We assess levels of functional play, symbolic play, and play diversity using the Free Play Protocol as per Laplante, et al. [49]. The children were given the opportunity to play with age-appropriate toys on their own for 10 minutes and were videotaped for later coding and analysis.

##### Emotional availability and attachment relationship (16 months and 2½ years)

Emotional availability [146] is a standardized global measure of the nature of the mother-infant relationship. Mothers and infants engaged in a 10-minute joint play session, a ~3-minute separation, and a 5-minute reunion play session. Mother-child interactions were videotaped for later coding and analysis. Four dimensions of maternal qualities are being coded: sensitivity, structuring, non-intrusiveness, and non-hostility. Two infant qualities are also being coded: responsiveness to the mother and involvement with the mother. The validity and stability of the construct over time have been established [147] and the data can give information about the nature of the relationship between the dyad, in terms of a ‘secure’ or ‘insecure’ attachment relationship.

##### Parental control and rejection (4 years)

Children will be provided with two different and challenging tasks (e.g. jigsaw puzzle, speech task), suitable for children between 5 to 6 years of age such that these tasks would normally be beyond their ability to solve alone. The child will be instructed to complete each task in five minutes while their mother provides support nearby. The task will be video recorded and maternal behavior will be coded for control and rejection, two theoretical concepts that have been implicated in the development of anxiety in children. As per Hudson and Rapee [148], maternal behavior will be rated on 10 global scales, scored on a 9-point continuum from 0 to 8 with 4 being neutral. The 10 scales assess intrusiveness, helpfulness, physical involvement, maternal focus, posture, positive affect, mood, tension, encouragement/criticism, and child’s response to mother [148].

#### Biological samples: mother, birth, and child

##### Maternal pregnancy blood samples (36 weeks gestation)

Women still pregnant at the time of recruitment had additional blood drawn for the purposes of QF2011 at the time of routine antenatal blood tests collected at 36 weeks gestation. An additional 4 × 5ml vials were drawn, centrifuged, then stored at -80°C (plasma and serum). Blood samples will be analyzed for (a) endocrine markers; (b) immune panel (cytokines); and (c) biomarkers of nutrition panel.

##### Maternal diurnal cortisol (recruitment and 12 months post-flood)

At each time point, participants were asked to collect saliva samples over two days at the following times: waking (w), w + 30 min, w + 45 min, w + 60 min, and before bed, taking note of all times and, if applicable, times of breastfeeding before and during sampling [149]. Cortisol concentrations are determined by competitive enzyme immunoassay (EIA) using Salimetrics kits [150, 151].

##### Maternal DNA (16 months, or 2½ years, or 4 years)

Mothers provided a saliva sample for the purposes of genotyping using Oragene DNA OG-500 kits. Saliva for DNA can be stored at room temperature for several years before processing due to the presence of a chemical preservative in the collection tube provided. Women provided saliva for DNA at the 16 month or the 2½ year assessment. If necessary, saliva samples for DNA will be collected at the 4 year assessment from women who had not provided samples previously.

##### Placenta

The placenta was collected within 20 minutes after expulsion then sampled and processed within 60 minutes. Each placenta was inspected (morphology, weight, and cord position, calcification, etc.) before sampling. Chorionic villi biopsies were collected as per a standardized protocol proposed by the International Federation of Placenta Associations [152]. Multistage unbiased random sampling (giving all parts an equal chance of being chosen) of eight pieces, each one 1 cm^3^, was conducted from the fetal side of each placenta to avoid maternal decidual and fetal amnion/chorion membrane contamination [153, 154]. Placental villi samples were snap-frozen then stored at -80°C. Fetal chorion/amnion membrane, decidua, and chorionic villi were also sampled from two sites (center and perimeter) of the placenta, rinsed of blood, snap-frozen, and stored at -80°C.

##### Umbilical cord

The total length of the umbilical cord was measured, and five 1 cm pieces of umbilical cord (in the sterile area) were collected, snap frozen and stored at -80°C.

##### Cord blood

Cord blood was collected following expulsion of the placenta. The umbilical cord was clamped off from the newborn and blood samples were collected from the umbilical vein using a syringe, then transferred to both serum-separating and heparin tubes. Serum and plasma were collected from these tubes, respectively (around 5-10 mL each) and stored at -80°C.

##### Neonatal reactive cortisol (2-4 days postpartum)

Saliva samples were collected from neonates to assess HPA reactivity to a stressor: the routinely administered, but painful, heel prick procedure used to screen neonates for a range of metabolic and other conditions. Samples were taken using Salimetrics SalivaBio Swabs or Salimetrics Cotton Dental Swabs at three time points: immediately before the heel prick (baseline), and 20 and 40 minutes post-heel prick. As per the manufacturer’s instructions the Bioswabs were centrifuged immediately at 3000-3500 rpm for 15 min.

##### Child diurnal cortisol (16 months and 2½ years)

Saliva samples were collected from the children using Salimetrics Children’s Swabs (Item number 5001.06). Mothers were asked to take a total of six saliva samples from their child at waking, 30 minutes after waking, and at bedtime for two consecutive days. Samples were stored in the family refrigerator before being brought in for the testing session where the samples were then stored at -80°C. Concentrations of diurnal salivary cortisol were determined by competitive enzyme immunoassay (EIA) using kits provided by Salimetrics.

##### Child reactive cortisol (16 months and 2½ years)

As noted earlier in the description of the child’s behavioral stress reactivity, there was a mother-child separation during the joint play sessions, during which the mother left her child alone in the room for a period of up to three minutes while being monitored by the mother and research assistant by a one-way mirror. To assess changes in cortisol levels, child saliva was collected using Salimetrics Children’s Swabs immediately before the separation, and 20 minutes after the end of the separation [155].

##### Child testosterone (16 months and 2½ years)

Saliva samples for testosterone were obtained using Salimetrics Children’s Swabs (Item number 5001.06). Mothers collected the sample in the morning and kept them in the family refrigerator. Mothers brought the samples into the lab on assessment day where they were stored in a -80°C freezer. Samples were assayed in duplicate using a highly sensitive enzyme immunoassay.

##### Child DNA samples (2½ years)

Saliva for genotyping was obtained using Oragene DNA collection kit OG-575. This non-invasive collection technique involves placing a sponge into the child’s cheek pouch and gently rubbing it against the child’s gums and inner cheek for about 30 seconds. Once the sponge is saturated with saliva, it is inserted into the V-notch of the collection vial and twisted to allow the saliva to flow downwards. This procedure is repeated until 0.75 mL of saliva is obtained. This procedure yields approximately 17.3 ug of DNA. If necessary, due to missed samples at age 2½, saliva samples for DNA will be collected at the 4 year assessment.

#### Routine database and chart audit

##### Mother’s chart

We requested the extraction of 273 variables from ‘Matrix’, the Mater Mother’s Hospital database. These variables include (but are not limited to): maternal height and pre-pregnancy weight (BMI); all medications prescribed during pregnancy; blood test reports from routine visits (first visit, 16-20 weeks (optional), and 26-28 weeks (incl. glucose tolerance)); and obstetric outcomes.

##### Ultrasound reports

We will obtain any existing ultrasound reports for biometry and growth, blood flow to uterus (uterine arteries), fetus (umbilical vessels), and within the fetus (cerebral arteries). Of particular interest will be the crown-rump length, biparietal diameter, occipitofrontal diameter, and cephalic index, femur and humerus length, head circumference, abdominal circumference, estimated fetal weight, estimated due date, ultrasound gestational age, amniotic fluid index +/- deepest vertical pocket, and umbilical artery Doppler.

##### Baby’s chart

Chart review was performed to collect data on infant sex, as well as birth outcomes including birth weight, length and head circumference, gestational age at birth, one and five minute APGAR scores, and any newborn complications (respiratory distress, infection, congenital anomalies, hypothermia).

#### Data analysis and power

Our analyses of the QF2011 data will proceed in a highly structured manner, according to our underlying explanatory model. This model posits that objective stress and cognitive appraisal predict peritraumatic stress responses, which predict both subjective stress reaction (PTSD symptoms) and maternal HPA axis response. We hypothesize that at least some of these aspects of maternal stress may influence maternal mental health and may also influence variables within the placenta and fetus (cord blood); thereby influencing fetal/child physical/physiological development (growth & body composition, fingerprints or finger length ratios, HPA axis) and child neurodevelopment (cognitive, behavioral, and motor development). We further suggest that at each level of the cascade there may be genetic moderation and epigenetic mediation; and also moderation of outcomes by pre-disaster maternal characteristics (e.g., depression, socioeconomic status), and by the sex of the infant. Finally, our results may indicate a moderating effect from prenatal care (MGP versus standard care) and maternal caretaking behaviors. Below, we present a sampling of statistical approaches we will take to the data analysis.

##### Maternal short-term response to the floods

Our first task is to understand the associations among the psychosocial and biological responses of the mother to the stress of the flood. Associations among objective stress (QFOSS), cognitive appraisal, peritraumatic distress (PDI) and dissociation (PDEQ), PTSD symptoms (IES-R), and maternal diurnal cortisol will be explored using correlations, and trend analyses to determine if any of the associations are non-linear. We will also test the ability of maternal genes associated with the HPA axis to moderate the effects of flood stress on maternal diurnal cortisol after the floods.

##### Maternal mental health

Next, we will examine genetic and psychosocial mediators and moderators of the associations between flood stress and 12-month post-flood maternal outcomes such as symptoms of anxiety (STAI) and PTSD, as well as positive mental health (MHC-SF). We will test the ability of maternal coping style (Brief COPE) and specific candidate genes to moderate the effects of objective stress exposure on mental health outcomes. For women who had the EPDS administered before the floods, we will also determine the moderating effects of pre-flood depression (as measured by the EPDS) in the association between objective stress exposure and maternal mental health outcomes. Our general approach is to use hierarchical multiple linear regression, adding variables into the equation one block at a time. We first regress maternal mental health on exogenous variables such as maternal genotype and demographics; next, we add objective stress exposure; peritraumatic distress and dissociation; then psychosocial variables needed for the interaction (e.g., coping style); and finally we test one interaction at a time (e.g., objective stress-by-coping). We also test moderation and mediation effects directly using the SPSS PROCESS command; the mediation analyses include bootstrapping.

##### Birth biologicals

These analyses will be guided by our models of the MPF axis, the first involving a cascade of effects via maternal cortisol to the child’s HPA axis functioning (diurnal and reactive cortisol), and second involving a cascade through the catecholamine/inflammatory system. We will begin by testing bivariate associations throughout the model, culminating in structural equation modelling, including testing of moderation of effects by fetal sex and by timing in gestation of the floods.

##### Child development

We will again use hierarchical linear multiple regression to test the effects of PNMS on child physical, cognitive, behavioral and motor development at each age, testing models similar to those described above for maternal mental health. For those variables that will be assessed at multiple time points (e.g., Ages and Stages, and CBCL), we will employ multi-level modelling once all of the data are obtained at age 4 years. This approach will determine the effects of PNMS on the initial measures of child functioning, and then on the slope of change in functioning over time.

##### Power

Our best estimates of effect sizes, and feasibility, come from Project Ice Storm which began with approximately 160 live births in 1998. Sixteen years later, there are still 75 families taking part in assessments. With this cohort, we have had sufficient statistical power to show significant effects of the severity and/or timing of ice storm stress on: gestational age, obstetric complications, IQ, language, memory, attention, internalizing and externalizing problems, autistic-like symptoms, fingerprint asymmetry, obesity, brain structure, and other outcomes. Although Project Ice Storm lacks the sample size to test important interactions and advanced modelling, these data nonetheless suggest that we have adequate statistical power with respect to the QF2011 study.

The QF2011 study noted an initial formal withdrawal rate of 15.9 % between recruitment and the first face-to-face assessments of infants at 16 months of age, the withdrawal rate at the 2½ year assessment dropped to 3.7 %. Moreover, of the 191 families remaining in the study at 16 months, 91.6 % completed some portion of the assessment. At age 2½ years, 175 families provided data. Currently, a sample of 209 families can be contacted for the 4 year assessment which began in 2015. Using a conservative participation rate of 80 %, it is estimated that data will be available for approximately 167 families at this assessment.

### Ethics approval

Ethics approval for the initial protocol was received from the Mater Hospital Human Research Ethics Committee (1709M) on 4^th^ April 2011; subsequent protocols were approved for the 16 month, 2½, and 4-year face-to-face assessments (Ref. #1844M). The study also has approval from The University of Queensland (2013001236).

## Discussion

Two 2011 *Nature* papers show that the production of greenhouse gases is responsible for the ever increasing risk of flooding [156, 157]. Prenatal stress research suggests that severe weather events increase the risk for adverse effects in the offspring, via fetal programming, that convey a huge public health burden. The goal of designing interventions for pregnant women in disasters or other stressful situations, to circumvent the effects of stress on the fetus, requires better understanding of the MPF mechanisms involved. The QF2011 study will increase current knowledge about the cascade of effects from an external event to the mother, through placenta and cord, to a host of outcomes in the unborn child. The pre-flood psychological assessments, biological samples at birth, and the embedded midwifery RCT examining the effect of Midwifery Group Practice, have provided a unique opportunity to compile the most comprehensive prospective data set ever obtained to study the effects of an independent stressor on child-bearing women, and risks for PTSD, postpartum depression, pregnancy outcomes, and infant development. By obtaining a multitude of maternal, placental and infant biomarkers from a large sample of women exposed to the flood at different points during pregnancy, we can bring the entire human maternal-placental-fetal system into the laboratory, and test integrated biopsychosocial models of prenatal stress for the first time. Armed with a better understanding of the processes involved, intervention strategies to prevent adverse outcomes in both mother and child can be designed and tested.

## Additional file

Additional file 1:
**Scoring of the Queensland Flood Objective Stress Scale (QFOSS).**

## Abbreviations

11β-HSD: 11β-hydroxysteroid dehydrogenase
2D:4D: The ratio of the lengths of the second (index) and 4th (ring) fingers
5-HT: 5-Hydroxytryptamine (Serotonin)
5HT2A: Serotonin receptor 2A
5-HTTLPR: Serotonin-transporter-linked polymorphic region
APGAR: Appearance, Pulse, Grimace, Activity, Respiration
ASQ: Ages and stages questionnaire
ASRS: Autism spectrum rating scales
BITSEA: Brief infant-toddler social emotional assessment
BMI: Body mass index
CBCL: Child behavior checklist
CES-D: Centre for epidemiologic studies depression scale
CIHR: Canadian institutes of health research
CRH: Corticotropin-releasing hormone
CRHR1: Corticotropin-releasing hormone receptor 1
CTQ: Childhood trauma questionnaire
C-TRF: Caregiver-teacher report form
DASS: Depression anxiety stress scales
DNA: Deoxyribonucleic acid
DOHaD: Developmental origins of health and disease
EPDS: Edinburgh postpartum depression scale
GC: Glucocorticoid
GLUT: Glucose transporter
HPA: Hypothalamic-pituitary-adrenal
hPL: Human placental lactogen
IES-R: Impact of event scale - revised
IQ: Intelligence quotient
ISAAC: International study of asthma and allergies in childhood
K-CPT: Conner’s kiddie continuous performance test
LES: Life experience survey
M@NGO: Midwives @ new group practice options
MCDI: MacArthur-Bates communicative development inventory
MGP: Midwifery group practice
MHC-SF: Mental health continuum – short form
MINI-Plus: Mini-international neuropsychiatric interview-plus
MMH: Mater Mothers’ Hospital
MPF: Maternal-placental-fetal
NART: National adult reading test
NEPSY: Developmental NEuroPSYchological assessment
OR: Odds ratio
PAFAS: Parenting and family adjustment scale
PDEQ: Peritraumatic dissociative experiences questionnaire
PDI: Peritraumatic distress inventory
pGH: Placental growth hormone
PNMS: Prenatal maternal stress
PPVT: Peabody picture vocabulary test
PSI: Parenting stress index
PTSD: Post-traumatic stress disorder
QFOSS: Queensland flood objective stress score
RCT: Randomized controlled trial
SCAS: Spence childhood anxiety scale
SSQSF: Social support questionnaire (Short Form)
STAI: State-trait anxiety inventory
STSI: Short temperament scale for infants
STST: Short temperament scale for toddlers
Th1/Th2: T-Helper 1/ T-Helper 2
THQ: Trauma history questionnaire
TOM: Theory of mind
VMI: Beery-Buktenika developmental test of visual motor integration
WPPSI-III: Wechsler preschool primary intelligence scale - version-III
