# Supplementary material for: QF2011: a protocol to study the effects of the Queensland flood on pregnant women, their pregnancies, and their children's early development
Source: BMC Pregnancy Childbirth. 2015 May 6;15:109. doi: 10.1186/s12884-015-0539-7 (PMC4518637; doi:10.1186/s12884-015-0539-7)
Supplement: Additional file 1: — Scoring of the Queensland Flood Objective Stress Scale (QFOSS). [file 12884_2015_539_MOESM1_ESM.docx]

Appendix 1 – Scoring of the Queensland Flood Objective Stress Scale (QFOSS)

|  | **Variable name** | **Variable Label** | **score** | **Value label** |
| --- | --- | --- | --- | --- |
| **Scope** | | | | |
| 1 | BackHomeDY | Were you unable to get to your home? If yes, for how many DAYS? | 5 | 0="Didn't leave home", 1='less than a day', 2='2 days', 3='3-4 days', 4='5-6 days', 5='7-16 days' |
| 2 | LeaveHome | Did the authorities advise you to leave your home because of the flooding? | 2 | 0='No', 2='Yes' |
| 3 | LeaveHomeDY | How many days did you ever leave your home because of the flooding? | 10 | 0="Didn't leave", 1='Less than half a day', 2='1-4 days', 3='5-8 days', 4='9-16 days', 5='21 days', 7='63 days', 8='79-83 days', 9='100-171 days', 10='249-364 days' |
| 4 | CircleDamage | How many people in your circle of family and friends had their home damaged because of the flooding? | 4 | 0='Null', 1='1-5 ppl', 2='6-10 ppl', 3='11-20 ppl', 4='21-41 ppl'. |
| 5 | Electricity | How many days did you lose electricity because of the flooding? | 6 | 0='No lose', 1=' Less than a day', 2='1-3 days', 3='4-7 days', 4='8-14 days', 5='15-21 days', 6='22-210 days' |
| 6 | HomePhone | How many days did you lose Home phone service because of the flooding? | 3 | 0='No lose', 1=' Less than 2 days', 2='2-6 days', 3='7-180 days' |
| 7 | Internet | How many days did you lose internet service because of the flooding? | 2 | 0='No lose', 1='Less than a week', 2='More than a week' |
| 8 | Mobile | How many days did you lose mobile service because of the flooding? | 3 | 0='No lose or less than a day', 1='1 day', 2='2-3 days', 3='4-5 days' |
| 9 | Petrol | How many days did you lose access to petrol because of the flooding? | 2 | 0='No lose', 1='Less than a week', 2='More than a week' |
| 10 | DrinkableWater | How many days did you lose drinkable water supply because of the flooding? | 5 | 0='No lose', 1='Less than 2 days', 2='3-4 days', 3='5-8 days', 4='14 days', 5='60-70 days' |
| 11 | Rubbish | How many days did you rubbish collection because of the flooding? | 2 | 0='No lose', 1='Less than 2 weeks', 2='more than 2 weeks' |
| 12 | Community | To what extent was your immediate community changed because of the flooding? | 6 | 1='A little', 0="Not at all", 2='Somewhat', 4='A lot', 6='To a great extent' |
| total |  |  | 50 |  |
| **Change** | | | | |
| 1 | ChangeHome | How many times were you required to change home because of the flood? | 6 | 0="None", 1='Once', 2='Twice', 4='3 times', 6='4 times' |
| 2 | NewHome | Have you moved into a new permanent home because of the flood? | 3 | 3='Yes', 0='No' |
| 3 | HouseAdult | How many adults did you house in your home during the flooding? How many days? | 4 | 0='No', 1='ppl # * Days: less than 7', 2='ppl # * Days: 8 -14', 3='ppl # * Days: 15-30', 4='ppl # * Days: 31-720'. |
| 4 | HouseChild | How many children did you house in your home during the flooding? How many days? | 4 | 0='No', 1='ppl # * Days: less than 7', 2='ppl # * Days: 8 -14', 3='ppl # * Days: 30-60', 4='ppl # * Days: 61-72' |
| 5 | WorkPlaceDamage | Was your place of work, or the company for whom you work, damaged by the flooding? | 1 | 0='No, not damaged' 1 'Yes, damaged' |
| 6 | WorkHour | Did you experience a change in your work hours because of the flooding? | 1 | 0='Same overall number of hours and my schedule stayed the same', 1='schedule changed, or change in work hours' |
| 7 | WorkHourp | Did your partner experience a change in work hours because of the flooding? | 1 | 0='No', 1='Yes' |
| 8 | LaidOffDY | Were you temporarily laid off from your job because of the flooding? How MANY DAYS? | 2 | 0 =NA', 1='Less than 7 days', 2='7-14 days' |
| 9 | LaidOffDYP | Were your partner temporarily laid off from your job because of the flooding? How MANY DAYS? | 1 | 0='NA', 1='1-60 days or Still not returned' |
| 10 | LoseJob | Did you lose your job permanently because of the flooding? | 5 | 0='No', 5='Yes' |
| 11 | LoseJobP | Did your partner lose their job permanently because of the flood? | 3 | 0='No', 3='Yes' |
| 12 | PartnerSeparate | How many days were you separated from only your partner? | 5 | 0='NA', 1='1-2 days', 2='5-7 days', 3='10 days', 4='21 days', 5='30 days'. |
| 13 | OtherSeparate | How many days were you separated from other member(s) of your household? | 2 | 0='NA', 1='1-7 days', 2='21 days' |
| 14 | PhysicalActivity | Did you experience a change in your level of physical activity because of the flooding? | 1 | 0='No', 1='Yes' |
| 15 | CommuteTime | Did you experience a change in your commuting times (in minute) because of the flooding? | 2 | -1='Shorter', 0='NA', 1='Longer, Less than 60 mins', 2='Longer, more than 60 mins' |
| 16 | AntenatalCare | Did you experience difficulty in accessing antenatal care because of the flooding? | 1 | 0='No', 1='Yes' |
| 17 | BrithPlan | Did you make any changes to your birth plan because of the flooding? | 1 | 0='No', 1='Yes' |
| 18 | PostnatalCare | Did you experience difficulty in accessing postnatal care because of the flooding? | 1 | 0='No', 1='Yes' |
| 19 | DietBackNormal | If you experience a change in your diet due to flooding, when did your diet return to 'normal'? | 2 | 0='No change', 1='After 1-7 days', 2='After more than one week' |
| 20 | CareOutsideLevel | If you were responsible for the care of anybody outside of your immediate household during the flooding, did your level of care for these people change because of the flooding? | 4 | 0='No change to my level of care', 1= 'Yes - my level of care for these people increase, house 1-2 ppl' , 2='Yes - my level of care for these people increase, house 3-4 ppl' , 3='Yes - my level of care for these people increase, house 12 ppl', 4='Yes - my level of care for these people increase, house 30 ppl' |
| total |  |  | 50 |  |
| **Loss** | | | | |
| 1 | InsurVSPrimaryHome | Primary home damage VS insurance | 8 | 0='Untouched by the flooding', 2='Damaged but able to live there, has insurance',  4='Damaged but able to live there, no insurance OR Totally destroyed, has insurance', 3='Damaged and unable to live there, has insurance', 6='Damaged and unable to live there, no insurance', 8='Totally destroyed, no insurance' |
| 2 | InsurVS2ndHome | Second home damage VS insurance | 5 | 0='Untouched by the flooding', 1='Damaged but able to live there, has insurance', 2='Damaged but able to live there, no insurance OR Damaged and unable to live there, has insurance', 3='Totally destroyed, has insurance', 4='Damaged and unable to live there, no insurance', 5='Totally destroyed, no insurance' |
| 3 | InsurVSVehicle | Vehicle dmaage VS insurance | 3 | 0='No damage', 1='Slightly damaged, no insurance OR moderately damage, has insurance', 2='Moderately damaged, no insurance OR total loss, has insurance' , 3='Total loss, no insurance' |
| 4 | InsurVSMaterialLoss | Material loss and damage VS insurance | 8 | 0='Loss $0', 1='Loss up to $100 OR Loss $101-$1000, 100% covered', 2 'Loss $1001-$10,000, 100% covered OR Loss $101-$1000, less than 50% covered', 3='Loss $1001-$10,000, less than 50% covered', 4='Loss $10,001-$100,000, 100% covered', 5='Loss $10,001-$100,000, 25-50% covered OR Loss $100,001-$200,000, 100% covered', 6='Loss $10,001-$100,000, 25-50% covered OR Loss $100,001-$200,000, 100% covered', 7='Loss $100,001-$100,000, less than 10% covered OR Loss more than $200,000, 25-50% covered', 8='Loss more than $200,000, less than 10% covered' |
| 5 | InsurVSBusiLoss | Personal business loss VS insurance | 8 | 0='No personal business', 2='Loss $101-$1000, no insurance', 3='Loss $1001-$10,000, no insurance' |
| 6 | InsurVSBusiLossP | Partner's personal business loss VS insurance | 8 | 0='No personal business' , 2="Loss $101-$1000, no insurance", 3='Loss $1001-$10,000, no insurance' , 4="Loss $10,001-$100,000, 100% covered", 5="Loss $100,001-$200,000, 100% covered", 6="Loss $100,001-$200,000, 50% covered", 7="Loss $100,001-$200,000, no insurance" |
| 7 | Income | Did you experience a loss of personal income because of the flooding? | 3 | 0='No', 3='Yes' |
| 8 | IncomeP | Did your partner experience a loss of personal income because of the flooding? | 3 | 0='No', 3='Yes' |
| 9 | Heirloom | Loss of precious items and/or heirloom? | 2 | 0='No', 2='Yes' |
| 10 | Property | Loss of personal property other than precious items and/or heirloom? | 2 | 0='No', 2='Yes' |
| total |  |  | 50 |  |
| **Threat** | | | | |
| 1 | Strand | How many days were you stranded in your home and unable to get out? | 5 | 0='Null', 1='1 day', 2='2 days', 3='3 days', 4='4 -7 days', 5='56 days' |
| 2 | NUMWarning | Total number of warning received | 2 | 2='0-1 warning', 1='2-3 warnings', 0='4-7 warnings' |
| 3 | DYWarning | How long before the flooding did you receive your first warning? | 2 | 2='Less than 1 day', 1='1-3 days', 0='4-7 days' |
| 4 | LeaveTime | How much time were you given before you were required to leave your home? | 4 | 4 ='Less than 6 hours', 3='7 - 12 hours', 2='13 - 24 hours', 1='More than 1 day', 0='Not required to leave'. |
| 5 | Hurt | Were you physically hurt because the flood? | 3 | 0='No', 1='Mildly Hurt' |
| 6 | CircleHurt | In your circle of family and friends, was anyone physically hurt because the flood? SUM (mild injury, moderate injury*2, severe injury*3) | 3 | 0='Null', 1='1 mild injury', 2='2 mild injury or 1 moderate injury', 3='composite score 3-8'. |
| 7 | CircleHurtWitness | How many injuries in your circle of family and friend did you witness? | 3 | 0='Null', 1='witness mild injury', 2='4 mild and 2 moderate injury happened, did not specify which injury she witnessed', 3='witness severe injury'. |
| 8 | OutCircleHurtWitness | Other than people in your circle of family and friends, how many people (i.e., strangers) did you witness being injured:SUM (mild injury, moderate injury*2, severe injury*3) | 4 | 0='Null', 1='1 mild injury', 2='mild injury or 1 moderate injury', 3='composite score 3-6', 4= 'composite score 26' |
| 9 | ThinkDie | Did you think you were going to die at any time during the flooding? | 7 | 0='No', 7='Yes' |
| 10 | CircleDie | In your circle of family and friends, did anybody die because of the flood? | 5 | 0='No', 5='Yes' |
| 11 | OutCircleDie | Other than people in your circle of family and friends, did you personally witness the death of somebody because of the flood? | 5 | 0='No', 5='Yes' |
| 12 | NUMDanger | The total number of situations of danger your life was in | 4 | 0='0', 1='1', 2='2', 3='3', 4='4' |
| 13 | DYDanger | The total amount of DAYS that you were in danger | 3 | 0='Not in danger', 1='Up to a day', 2='More than 1 day and less than a week', 3='7-31 days'. |
| total |  |  | 50 |  |
